# A single infusion of intravenous lidocaine for primary headaches and trigeminal neuralgia: a retrospective analysis

**DOI:** 10.3389/fneur.2023.1202426

**Published:** 2023-08-10

**Authors:** C. F. Mullins, M. Fuccaro, D. Pang, L. Min, A. P. Andreou, G. Lambru

**Affiliations:** ^1^The Headache and Facial Pain Service, Guy’s and St Thomas’ NHS Foundation Trust, London, United Kingdom; ^2^Pain Management and Neuromodulation Centre, Guy’s and St Thomas’ NHS Foundation Trust, London, United Kingdom; ^3^King’s College London, Institute of Psychiatry, Psychology and Neuroscience, London, United Kingdom

**Keywords:** intravenous lidocaine, migraine, SUNCT, SUNA, cluster headache, trigeminal neuralgia, refractory headaches, transitional treatments

## Abstract

**Introduction:**

Intravenous (IV) lidocaine has been used as a transitional treatment in headache and facial pain conditions, typically as an inpatient infusion over several days, which is costly and may increase the risk of adverse effects. Here we report on our experience using a single one-hour IV lidocaine infusion in an outpatient day-case setting for the management of refractory primary headache disorders with facial pain and trigeminal neuralgia.

**Methods:**

This is a retrospective, single-center analysis on patients with medically refractory headache with facial pain and trigeminal neuralgia who were treated with IV lidocaine between March 2018 and July 2022. Lidocaine 5 mg.kg^−1^ in 60 mL saline was administered over 1 h, followed by an observation period of 30 min. Patients were considered responders if they reported reduction in pain intensity and/or headache frequency of 50% or greater. Duration of response was defined as short-term (< 2 weeks), medium-term (2–4 weeks) and long-term (> 4 weeks).

**Results:**

Forty infusions were administered to 15 patients with trigeminal autonomic cephalalgias (*n* = 9), chronic migraine (*n* = 3) and trigeminal neuralgia (*n* = 3). Twelve patients were considered responders (80%), eight of whom were complete responders (100% pain freedom). The average duration of the treatment effect for each participant was 9.5 weeks (range 1–22 weeks). Six out of 15 patients reported mild and self-limiting side effects (40%).

**Conclusion:**

A single infusion of IV lidocaine might be an effective and safe transitional treatment in refractory headache conditions with facial pain and trigeminal neuralgia. The sustained effect of repeated treatment cycles in some patients may suggest a role as long-term preventive therapy in some patients.

## Introduction

The core of the management of primary headache disorders and trigeminal neuralgia (TN) is long-term prevention. However, these conditions can present with recurrent pain attacks or periods of abrupt worsening of an otherwise long-standing chronic condition, with significant ictal and interictal disability, emergency department attendances and hospital admissions (1–5). Treatments with a fast onset of effect but with short therapeutic duration are often labelled as “transitional treatments.” Different treatment approaches, including medication dose manipulations, add-on short term oral preventive treatments, minimally invasive injectable treatments and infusions of certain medications have been attempted with mixed outcomes (6, 7). Furthermore, the tendency to poor treatments outcomes of a significant minority of primary headache and TN patients, often adds another degree of complexity in the management of these patients’ worsening phases (8).

The effectiveness of intravenous (IV) lidocaine as a continuous infusion in the abortive treatment of migraine with and without medication overuse, status migrainosus and chronic daily headache has been shown in several open-label case series (9–14). Among the TACs, sparse evidence is available for cluster headache (CH). Conversely substantial open-label evidence of effectiveness of continuous infusion of lidocaine is available for SUNCT/SUNA (15, 16). A recent meta-analysis of the published evidence of IV lidocaine as a transitional treatment in SUNCT/SUNA, showed excellent, although short-lived effect in 90% of cases reported (6). Small case series have suggested the effectiveness of IV lidocaine in TN (17, 18).

Intravenous lidocaine in chronic headache disorders has typically been administered as a hospital-based infusion over several days (9–12, 19–21). However, such prolonged hospital admissions are costly and associated with potential adverse effects including neuro-psychiatric and cardiac side effects (15, 16). The available evidence confirms the rapid onset of effect of this treatment in certain primary headache conditions and in TN. However, it is unclear how long the benefits of an infusion can last for and what is the sustained effectiveness over subsequent infusions over time. The European Academy of Neurology and the Royal College of Surgeons (RCS) England have given a weak recommendation for its use in TN with a recommended infusion dose of between 1.5–5 mg.kg^−1^.hr.^−1^ for 60 min (22). In this report we present our experience using a single 60-min IV lidocaine infusion in an outpatient day-case setting as a transitional treatment for treatment-resistant primary headache disorders with facial pain and trigeminal neuralgia.

## Materials and methods

A retrospective analysis on consecutive patients treated with IV lidocaine at Guy’s and St Thomas’ headache and facial pain service between March 2018 and July 2022 was undertaken. Patients met the International Headache Society (23) criteria for a primary headache disorders with facial pain or TN. The more recent International Classification of Orofacial Pain (ICOP) was also employed to ensure accurate subclassification of primary headache disorders with associated orofacial pain (24, 25). Patients who are offered this treatment have to be treatment-resistant as per consensus statements or clinical experience depending on the condition treated (24). All patients had a chronic sub-type of their condition, with daily attacks for the short-lasting conditions (SUNCT/SUNA, TN and CH) and with a daily headache pattern for the long-lasting conditions (migraine and HC). IV lidocaine was administered in our pain and headache day-case procedure suit, using a local agreed protocol which involved continuous cardiac and pulse oximeter monitoring and blood pressure monitoring every 5 min during the infusion. After obtaining informed consent and excluding the major contraindications to the treatment (i.e., history of significant cardiac disease, arrhythmia, seizures, and previous allergic reactions to lidocaine), patients were administered with lidocaine 5 mg/kg diluted in 60 mL saline and infused over 1 h and were then observed for 30 min for possible delayed adverse events, prior to being discharged. Preventive treatments were not changed before and after the IV lidocaine treatment effect was assessed. Patients were followed up 6 weeks later with a telephone follow-up appointment to establish the outcome of the therapy and arrange the following infusion if appropriate.

Baseline patient demographics, diagnosis, headache/facial pain characteristics and the number of previous failed therapies were recorded for each patient. Outcome data were collected retrospectively by reviewing the electronic patient records for subsequent patient encounters. All patients were called directly to confirm efficacy outcomes and adverse events and to collect missing data from the records. The main efficacy outcome was change in headache/facial pain intensity using a verbal rating scale (VRS) 0–10. Patients were considered responders if they reported a reduction in headache/facial pain intensity of at least 50% compared to baseline. Changes in frequency of attacks was also evaluated when available. For patients with short-lasting headache/facial pain conditions, change in daily attack frequency was assessed. For patients with long-lasting headache conditions, changes in headache days were evaluated. The same effectiveness outcomes were used for subsequent IV lidocaine treatments. Data were collected on adverse events; the presence, severity and type of side effects that occurred during the infusion were obtained from discharge letters, notes taken by nursing staff during the procedure and from follow-up telephone calls.

To calculate the fluctuation in response to treatment among participants, the standard deviation (SD) of individual participants’ treatment response was calculated and the average SD of participants was used. Statistical analysis was performed using SPSS version 28 (IBM, NY, United States). A log-rank test was used to compare treatment duration according to diagnosis. Statistical significance was set at *p* < 0.05. The study was an audit of outcome and hence no formal ethical review was therefore required.

## Results

Between March 2018 and July 2022, 15 patients were treated with IV lidocaine and a total of 40 infusions were undertaken (Table 1). Six patients had chronic SUNCT/SUNA, three had chronic orofacial migraine (as per ICOP criteria), three patients had TN (two secondary to multiple sclerosis and one idiopathic purely paroxysmal), two had chronic CH and one patient had hemicrania continua (HC). The average age at the first infusion was 50 years old (range 24–73). All patients’ conditions displayed a chronic pattern for an average of 14.9 years prior to the first IV lidocaine treatment. Co-existing CM and fibromyalgia were present in five patients.

**Table 1:** Patient characteristics and response to treatment categorized by diagnosis.

Diagnosis	Participant	Age (average)	Sex	Time since diagnosis, years	Number of preventative treatments trialed, average (SD)	Number of lidocaine infusions, *n* (average)	Immediate responders (> 50% pain relief), *n* (%)	Short term responder (< 2 weeks)	Medium term responder (2–4 weeks)	Long term responder (> 4 weeks)	Average percentage of relief, %	Average duration of benefit, weeks	Average treatment frequency, months
SUNCT/ SUNA	1	73	F	20	14	1	✓	✓			100%	1	-
	2	56	F	10	25	3	✓			✓	100%	6	4
	3	57	F	12	8	2	✓			✓	75%	22	13
	4	63	F	14	13	1	X				0%	0	-
	5	53	F	6	8	2	✓			✓	100%	22	17
	6	33	F	4	6	1	X				40%	5	-
*Subtotal*	*6*	*55.8*		*11.0*	*12.3 (6.9)*	*10 (1.7)*	*4 (67%)*	*1 (25%)*	*0 (0%)*	*3 (50%)*	*69%*	*11.2*	*11*
Chronic facial migraine	7	27	F	30	9	7	✓			✓	80%	6	6
	8	47	F	22	6	1	✓			✓	100%	8	-
	9	30	F	14	17	2	✓			✓	100%	17	5
*Subtotal*	*3*	*34.7*		*22.0*	*10.7 (5.7)*	*10 (2)*	*3 (100%)*	*0 (0%)*	*0 (0%)*	*3 (100%)*	*77%*	*9*	*4.5*
Trigeminal Neuralgia	10	66	M	16	5	4	✓			✓	60%	5	4
	11	57	F	9	8	2	✓			✓	70%	6	5
	12	24	F	2	0	1	✓			✓	100%	21	-
*Subtotal*	*3*	*49*		*9.0*	*4.4 (4)*	*7 (3)*	*3 (100%)*	*0 (0%)*	*0 (0%)*	*3 (100%)*	*93%*	*10.7*	*5.5*
CCH	13	48	M	10	20	10	✓		✓		100%	2	4
	14	57	M	15	16	2	✓			✓	50%	8	9
*Subtotal*	*2*	*52.5*		*12.5*	*18 (2.8)*	*12 (5)*	*2 (100%)*		*1 (50%)*	*1 (50%)*	*80%*	*5*	*6.5*
HC	15	55	F	11	18	1	X				0%	0	-
*Subtotal*	*1*						*0 (0%)*				*0%*	*0*	*0*
All	*15*	*50*		*14.9*	*11.5 (6.7)*	*40*	*12 (80%)*	*1 (8%)*	*1 (8%)*	*10 (83%)*	*83%*	*9.5*	*7.4*

SUNCT, short-lasting unilateral neuralgiform headache attacks with conjunctival injection and tearing; SUNA, short-lasting unilateral neuralgiform headache attacks with cranial autonomic symptoms; TN, trigeminal neuralgia; CCH, chronic cluster headache; MS, multiple sclerosis; HC, hemicrania continua; SD, standard deviation.

All patients had treatment-refractory conditions, having failed an average of 11.5 preventive treatments and 2.7 injectable or invasive treatments (Table 2). Eleven patients had failed more than one injectable treatment approach, most commonly botulinum toxin injection (*n* = 7), greater occipital nerve blocks (*n* = 5) and anti-calcitonin-gene related peptide (CGRP) monoclonal antibodies (*n* = 5). Invasive treatments failed included sphenopalatine ganglion (SPG) pulsed radiofrequency (*n* = 5), occipital nerve stimulation (*n* = 4), trigeminal microvascular decompression (*n* = 2), high cervical spinal cord stimulation (*n* = 2) and SPG stimulation (*n* = 2; Table 2). The single patient with HC had experienced long-term tolerability issues with indomethacin which resulted in treatment discontinuation and trials of other preventive treatments with some evidence in HC treatment failed to produce a meaningful improvement.

**Table 2:** Injectable or invasive treatments failed for each participant and categorized by diagnosis.

Diagnosis	Participant	Injectable or invasive treatments failed (average)	Botox	GON block	CGRP antibodies	SPG PRF	ONS	GGRF	MVD	SCS	SPG stimulation	Cryosurgery	Cervical spinal injections	Gamma knife
SUNCT/SUNA	1	3	✓		✓		✓							
	2	4	✓		✓	✓	✓							
	3	2				✓			✓					
	4	6	✓	✓				✓	✓			✓		✓
	5	1		✓										
	6	0												
*Subtotal*	*6*	*2.7*	*3*	*2*	*2*	*2*	*2*	*2*	*2*			*1*		*1*
Chronic facial migraine	7	2	✓	✓										
	8	2	✓		✓									
	9	4	✓	✓	✓								✓	
*Subtotal*	*3*	*2.7*	*3*	*2*	*2*								*1*	
Trigeminal Neuralgia	10	2						✓				✓		
	11	1						✓						
	12	0												
*Subtotal*	*3*	*1*						*2*				*1*		
CCH	13	4				✓	✓			✓	✓			
	14	4				✓	✓			✓	✓			
*Subtotal*	*2*	*4*				*2*	*2*			*2*	*2*			
HC	15	5	✓	✓	✓	✓							✓	
*Subtotal*	*1*	*5*	*1*	*1*	*1*	*1*							*1*	
*All*	*15*	*2.7*	*7*	*5*	*5*	*5*	*4*	*3*	*2*	*2*	*2*	*2*	*2*	*1*

SUNCT, short-lasting unilateral neuralgiform headache attacks with conjunctival injection and tearing; SUNA, short-lasting unilateral neuralgiform headache attacks with cranial autonomic symptoms; CCH, chronic cluster headache; HC, hemicrania continua; GON, greater occipital nerve; CGRP, calcitonin gene-related peptide; SPG PRF, sphenopalatine ganglion pulsed radiofrequency; ONS, occipital nerve stimulation; MVD, microvascular decompression; GG RF, Gasserian ganglion radiofrequency; SCS, spinal cord stimulation.

**Figure 1:**
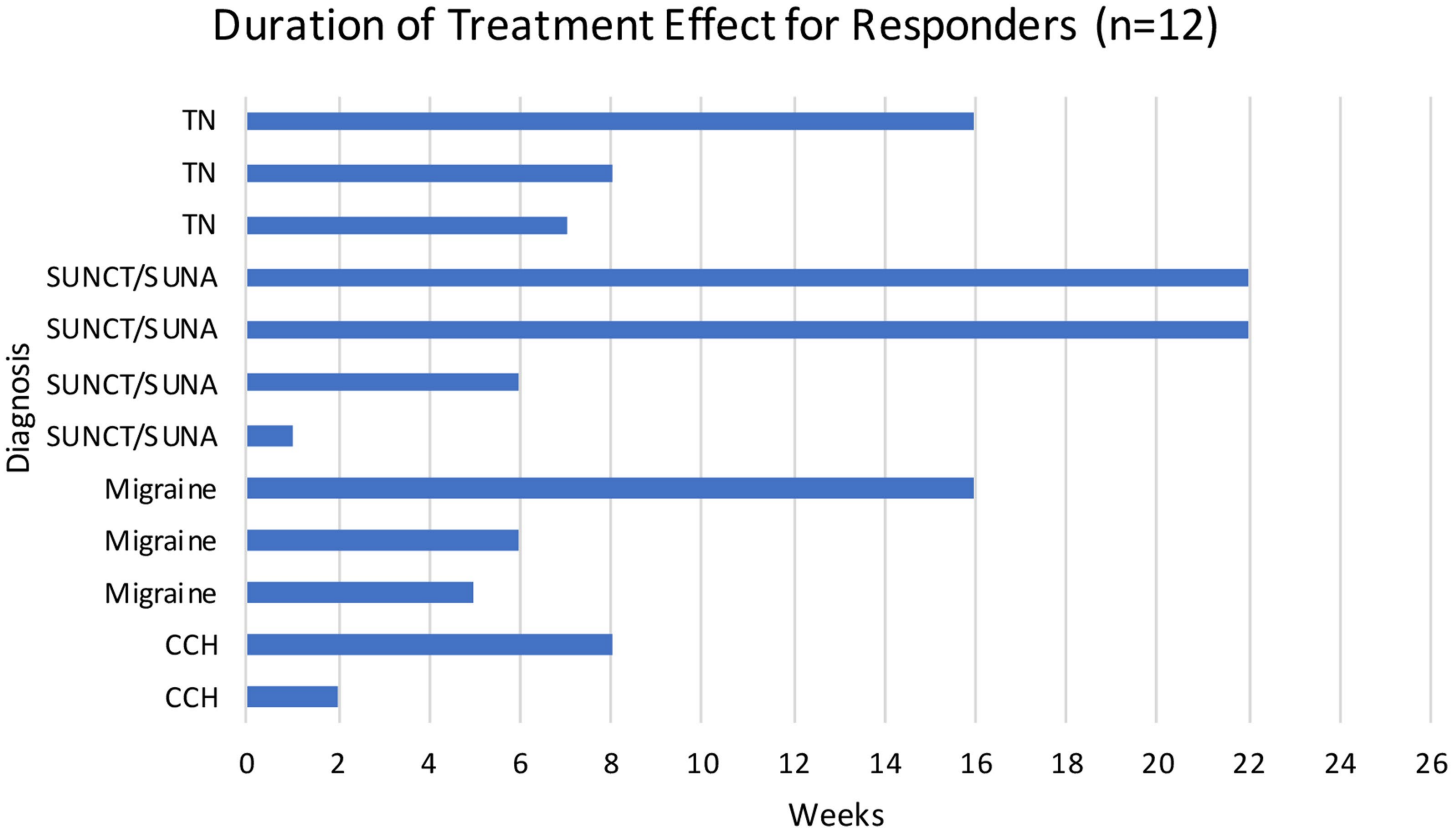
Duration of treatment effect for responders (*n* = 12).

Overall, of the 15 patients treated with one IV lidocaine infusion, 12 were considered responders (80%), eight of whom obtained a complete head/facial pain symptoms relief (53%). Of the remaining three, one was a partial responder (40% pain intensity reduction) and two were non-responders. The average duration of the treatment effect for responders was 9.5 weeks (range 1–22 weeks). Ten patients (83%) reported a duration of response longer than 4 weeks, one reported a duration of response of 3 weeks and one of less than 2 weeks (Figure 1). There was no statistically significant difference in treatment duration between patients’ diagnoses (Figure 1), *X*^2^ (1, *N* = 12) = 2.4, *p* = 0.495, however, our study was probably underpowered to detect a difference due to the small sample size. All responders were satisfied with the treatment.

Patients had an average of 2.7 infusion. Nine patients underwent more than one infusion (range 1–10). The average temporal interval between infusions was 7.4 months (range 1–17). The length of the interval between infusions was in some cases dictated by the suspension of the service during the COVID-19 pandemic. All responders to the first IV lidocaine treatment showed a sustained response to subsequent infusions with a SD of 9% in subsequent reported treatment response (range 0–35%). The SD of treatment duration for follow-up infusions was 4.1 weeks (range 0–18.4 weeks).

### Trigeminal autonomic cephalalgias

Six chronic SUNCT/SUNA patients underwent a total of 10 IV lidocaine infusions (Table 2). Four out of six patients responded to the treatment (67%). Besides the reduction in intensity, their daily frequency of attacks also reduced by 50% in responders. One patient was considered a partial responder and one did not respond. The average response duration was 11.2 weeks (range 1–22). Three patients had the treatment repeated at an average frequency of 11 months for reasons predominantly related to the COVID-19 pandemic. Two chronic CH patients underwent a total of 12 IV lidocaine infusions: one patient underwent 10 and one two infusions during the study period. Both were responders. One patient also experienced a reduction in daily attack occurrence of at least 50%. The average duration of benefit was 5 weeks and the treatment was repeated at an interval of 6.5 months. The HC patient did not respond to the treatment.

### Trigeminal neuralgia

Three TN patients underwent a total of 10 infusions. All three were responders with an average duration of response of 10.7 weeks (range 5–16 weeks). A reduction of at least 50% of the daily attacks was reported by all responders. Two patients had the treatment repeated during the study period at an average time interval of 5.5 months. One patient underwent seven infusions.

### Chronic orofacial migraine

Three patients with chronic orofacial migraine as per ICOP criteria underwent a total of six IV lidocaine infusions (25). All patients displayed a daily headache pattern at baseline. All three were considered responders displaying an average headache intensity reduction of 77% compared to baseline for an average duration of 9 weeks. Two patients had the treatment repeated at an average frequency of 4.5 months with one patient undergoing three infusions during the study period.

### Adverse events

Six out of 15 patients reported at least an adverse event during the infusion (40%). Two patients reported peri-oral tingling, light headedness and dull headache (different from their baseline condition). Limb numbness, transitory dysgeusia (spontaneous metallic taste), palpitations and tiredness were reported by one patient each. All side effects linked to the infusion were mild and self-limiting. Only one patient had the infusion temporarily stopped.

## Discussion

This retrospective analysis indicates that a short infusion of IV lidocaine administered in a day-case setting may be effective as a transitional treatment in treatment-refractory primary headache disorders with facial pain and TN. The vast majority of patients responded, regardless to whether their pain biology was neurovascular or neuropathic. Among responders, the majority became pain-free reporting 100% resolution of symptoms. It is noteworthy that our patients were treatment-refractory, with many of them failing CGRP antibody therapy and invasive approaches. The beneficial effect was achieved immediately after the infusion in all responders and lasted a reasonable amount of time, considering the pharmacology of lidocaine (26). Indeed, some patients experienced meaningful symptoms control for up to 5 months. All responders wanted to have the treatment repeated and had more than one infusion with similar benefit to the first one, suggesting a sustained effect overtime of IV lidocaine.

Compared to the traditional way of administering IV lidocaine described in the majority of the literature on this treatment, infusing IV lidocaine as a short infusion in a day-case setting has several advantages. Hospital admissions for a prolonged infusion lasting between 2 and 9 days are not required (9, 10, 12, 19–21). This significantly reduces costs related to hospital admission and intensive prolonged monitoring, besides freeing up hospital beds. Furthermore, a short IV lidocaine infusion may limit the severity of the adverse events related to prolonged exposure to IV lidocaine and hospital stays in general. Indeed, in a recent retrospective study on the effect of IV lidocaine in headache disorders via admission to hospital, 10% of their patients required treatment cessation because of adverse events (21). In our report, none of the patients had to discontinue the treatment because of adverse effects. Because of these advantages, an infusion of IV lidocaine can be repeated over time, providing that the clinical benefits are meaningful and reasonably long-lasting and the tolerability profile is favorable for patients.

There is significant heterogeneity in the literature regarding doses and duration of lidocaine infusions. A recent retrospective study of systemic lidocaine in neuropathic chronic pain states employed a relatively large dose of lidocaine (1,000 mg) administered over a 25-h period and hypothesized that a certain minimum dose is required for efficacy, but that a greater incidence of adverse effects may be observed with higher infusion rates (27). In contrast, our study involved a lower total dose of lidocaine but a much faster infusion rate (5 mg.kg^−1^.h^−1^), and therefore a greater peak plasma concentration. Other studies have found that the effects of IV lidocaine may be dose-related (28, 29). An RCT investigating systemic lidocaine titrated to different plasma concentrations in pain thresholds and allodynia observed that a positive effect was only noted with highest plasma concentration and that a certain minimum plasma dose may be required to achieve a therapeutic effect (29). Guidelines for the use of perioperative systematic lidocaine recommend an initial dose of up to 1.5 mg.kg^−1^ using the patient’s ideal body weight, given over 10 min and, following that no more than 1.5 mg.kg^−1^.h^−1^ for up to 24 h (30). Other guidelines for the use of lidocaine in neuropathic pain have recommended IV lidocaine at doses of 5 mg.kg^−1^ to 7.5 mg.kg^−1^ (27). There are no studies comparing short duration infusions to longer inpatient infusions in our patient population in terms of efficacy and duration and the numbers of patients involved and the heterogeneity of the studies makes comparisons difficult. Ideally, these should be compared in a prospective study to determine if differences exist.

The role of IV lidocaine in migraine is still debated. Prolonged infusions of IV lidocaine have shown to possibly have a role in medication overuse withdrawal in patients with CM and MOH (9, 31). The application of IV lidocaine in status migrainosus has been evaluated in a retrospective study which looked at IV lidocaine, either alone or in combination with dihydroergotamine or subcutaneous sumatriptan. A bolus of 1 mg/Kg of lidocaine followed by an IV lidocaine infusion of 2 mg/min for a duration of 2 days was used, with improvements noted among the majority of this heterogeneous cohort (19). More prolonged infusions of IV lidocaine in chronic daily headache (CDH) for up to 8.5 days have been employed with positive outcomes (10). Sparse evidence has looked at the role of recurrent IV lidocaine infusions in the prevention of treatment-refractory CM, and chronic orofacial migraine (21). In view of the response rate and duration of the improvement showed in our patients, it is possible that 3–4 monthly IV lidocaine infusions could provide a meaningful degree of symptoms control in the refractory migraine population.

Intravenous lidocaine may have a role in the management of the TACs and TN, as a transitional treatment, in view of its rapid onset of effect, which is required in view of the pattern of occurrence of these conditions. The most compelling open-label evidence on the role of IV lidocaine amongst these conditions were produced in SUNCT/SUNA (7, 32–34). Intravenous lidocaine seems to be effective in the majority of patients with this condition. The treatment protocol used was again an in-hospital multiple-day infusion. Our findings confirm similar outcomes when a 60-min single infusion protocol is used. Similar favorable outcomes were also found in the three TN patients, which does not surprise given the clinical and therapeutic similarities between SUNCT/SUNA and TN (35). Previous evidence using single infusions of IV lidocaine showed similar results to ours, suggesting that single IV lidocaine infusions could be an attractive treatment options for SUNCT/SUNA and TN severe exacerbations (17, 18, 32). It is noteworthy that two of our patients had secondary TN and one had idiopathic TN, suggesting that IV lidocaine may be effective regardless of the TN subtype.

The anti-nociceptive mechanisms of lidocaine are thought to be mediated through its reversible blockade of sodium channels which modulates peripheral and central sensitization, cutaneous allodynia and intracranial hypersensitivity and can contribute to the underlying pathophysiology of primary headache disorders and TN (33, 34, 36, 37). The efficacy of agents that block voltage-gated sodium channels in TN and SUNCT, such as carbamazepine, lamotrigine, oxcarbazepine, demonstrate that sodium channel dysregulation is important in these conditions (6). Animal models have demonstrated decreased ectopic activity of voltage-gated sodium channels, C fiber spontaneous firing and trigeminal evoked potentials after IVL infusion or transdermal administration (19, 38, 39). The mechanism behind the prolonged anti-nociceptive effects following a short infusion of lignocaine is, however, unclear. The lidocaine metabolite n-ethylglycine may exert a prolonged anti-nociceptive effect through competitive inhibition of the glycine transporter GlyT1, inhibiting the ectopic activation of sodium channels (40). GlyT1 is important in regulating extracellular glycine concentrations, and blockade of this transporter results in an increased concentration of glycine within the blood and cerebrospinal fluid. Prolonged anti-nociceptive effects which outlast the pharmacological duration of reversible conduction blockade has been observed in multiple previous studies of local anesthetic nerve blocks and epidural injections (41–43). Lidocaine also possesses anti-inflammatory properties which can attenuate neutrophil activation and reduce the serum concentration of several interleukins (IL-1, 6 and 8) and intracellular adhesion molecule-1, which are important in the transport of inflammatory mediators and can induce allodynia and acute and chronic hyperalgesia. It is possible that lignocaine can reverse the wind-up and sensitization of chronic pain signals involved in neuroplasticity, however, the underlying neural correlates remain poorly understood (44).

### Side effects

Common side effects reported in the literature include light-headedness, perioral numbness, tinnitus, agitation and headache (21, 45). Arrhythmias and hypotension have been reported with the use of IV lidocaine and its use in patients with a history of cardiac arrhythmias is controversial with cardiac monitoring advised (21). Central nervous system side effects, such as hallucinations or convulsions, or neuropsychiatric side effects such as paranoid ideation, agitation or depression, may be more common with elevated plasma levels or with a longer duration of infusions. Two of four patients in Matharu et al. had to discontinue the infusion due to paranoid or suicidal ideation where infusions were continued up to 7 days (15). In Williams et al., where infusions in 14 subjects lasted an average of 8 days, one patient reported feeling depressed, three reported vivid dreams and, one patient with a history of a seizure disorder had a seizure, which was rapidly terminated by phenytoin and the IV lidocaine infusion recommenced at a lower rate (9). No cardiac adverse events were reported in these studies. Side effects were noted in 6 out of 15 patients in our patients (40%), however, these were all mild and self-limiting, with no cardiac or major central nervous system or neuropsychiatric events, and only necessitated temporarily stopping the infusion in one case.

The strengths of this analysis include: the use of a 60-min infusion protocol of IV lidocaine to explore its immediate and long-lasting effectiveness; the sustainability of this treatment protocol over multiple infusions; the use of this treatment for a treatment-resistant group of patients, in whom there is a great unmet need for safe and effective treatments. There are also many limitations, including the retrospective design and small sample size. However the data was accurately checked prospectively with the patients over the phone, so there was no missing data. The open-label nature of the study may suggest that the promising treatment outcome were biased by a degree of placebo. However, the highly refractory nature of our patients’ conditions and the sustained response after multiple infusions indicate the lack of a significant placebo effect. Prospective randomized-control trials with a placebo arm are warranted to establish greater quality evidence for use of IV lidocaine in this cohort. While we investigated the impact of IV lidocaine on a mixed cohort of patient with facial pain including TN and primary headache disorders, we did not investigate the effect on other facial pain syndromes including idiopathic facial pain syndromes or other neuropathic forms of facial pain and therefore, its effect in these conditions is unknown.

## Conclusion

Our initial clinical experience suggests that patients with treatment-refractory primary headache conditions and cranial neuralgias may benefit from a single infusion of 5 mg.kg^−1^ IV lidocaine infused in 60 min as a day-case admission with ECG monitoring. The benefit was achieved immediately in responders, who constituted the majority of patients treated. The head or facial pain improvement lasted on average for over 2 months with some patients experiencing symptom control for over 5 months with one infusion. When repeated, the treatment provided the same degree of improvement, suggesting a sustained effect over time. Of note, most patients tolerated the treatment very well with a proportion of patients reporting mild and transient side effect only, suggesting that this treatment protocol was overall well tolerated.

A single infusion of IV lidocaine could be a more practical, cost-effective and better tolerated way of administering IV lidocaine compared to the multiple-day hospital admissions. This treatment strategy could be effective as a transitional treatment but also, in some treatment-resistant patients, as a long-term preventive treatment with infusions performed every 3–4 months.

## Data availability statement

The raw data supporting the conclusions of this article will be made available by the authors, without undue reservation.

## Ethics statement

Ethical review and approval was not required for the study on human participants in accordance with the local legislation and institutional requirements. Written informed consent for participation was not required for this study in accordance with the national legislation and the institutional requirements.

## Author contributions

CM and MF: contributed in data collection, data analysis and manuscript preparation. DP and LM: administered the treatment and contributed in the manuscript preparation. APA: contributed in the data analyses and contributed in the manuscript preparation. GL designed the audit, diagnosed all patients, contributed in the data analyses and manuscript preparation. All authors contributed to the article and approved the submitted version.

## Conflict of interest

DP has received sponsorship to attend professional meetings from Medtronic and Nevro Corp.

APA has received speaker honoraria, funding for travel, and honoraria for participation in advisory boards sponsored by eNeura, Allergan, Lilly and Novartis.

GL has received speaker honoraria, funding for travel, and honoraria for participation in advisory boards sponsored by Abbvie, TEVA, Lundbeck, Lilly, and Novartis. He has also received speaker honoraria and funding for travel from electroCore, Nevro Corp, and Autonomic Technologies Inc.

The remaining authors declare that the research was conducted in the absence of any commercial or financial relationships that could be construed as a potential conflict of interest.

## Publisher’s note

All claims expressed in this article are solely those of the authors and do not necessarily represent those of their affiliated organizations, or those of the publisher, the editors and the reviewers. Any product that may be evaluated in this article, or claim that may be made by its manufacturer, is not guaranteed or endorsed by the publisher.
